# Chemical Interaction of Hydrogen Radicals (H*) with Transition Metal Nitrides

**DOI:** 10.1021/acs.jpcc.3c04490

**Published:** 2023-09-06

**Authors:** Abdul Rehman, Robbert W. E. van de Kruijs, Wesley T. E. van den Beld, Jacobus M. Sturm, Marcelo Ackermann

**Affiliations:** Industrial Focus Group XUV Optics, MESA+ Institute for Nanotechnology, University of Twente, Drienerlolaan 5, Enschede 7522NB, Netherlands

## Abstract

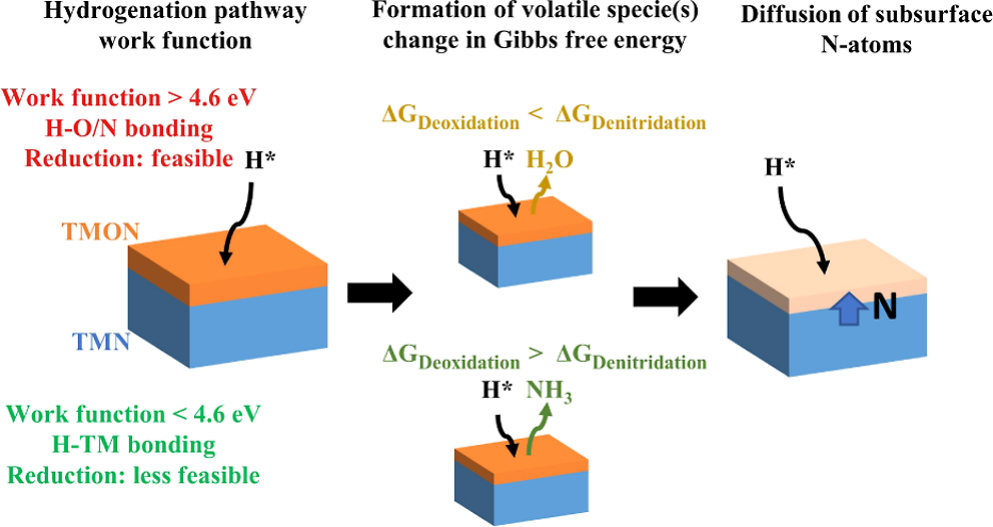

Transition metal nitrides (TMNs) are reported as protective coatings in reactive hydrogen environments. Although the permeation of H_2_ through TMN coatings is well reported, their reducibility in H* environments is less investigated. In this work, we categorize the interaction of H* with ambient exposed TiN, ZrN, HfN, VN, NbN, and TaN thin films at 700 °C into three classes. We find that in TiN and VN samples, H*-induced reduction was limited to the surface (≈ top 2 nm). Significant denitridation was observed in ZrN and HfN samples beneath the surface, along with an increase in the transition metal oxide (TMO_*x*_) fraction. Denitridation was observed in NbN and TaN samples as well, but the increase in the TMO_*x*_ content was less than for ZrN and HfN. We propose a model in three steps: hydrogenation, formation of volatile species, and diffusion of subsurface atoms to the surface. We show that the interaction of H* with TiN, ZrN, HfN, VN, NbN, and TaN with partially oxidized surfaces can be explained using the preferred hydrogenation pathway (based on the work functions) and the thermodynamic driver for forming volatile species (NH_3_ and H_2_O; based on the change in Gibbs free energy).

## Introduction

Hydrogen is an essential component in many technological applications, varying from energy production, transport, and storage, to usage as a process gas and/or surface reactant in various industries. For example, in fusion reactors,^1−3^ fuel cells,^4,5^ and aerospace applications,^6^ hydrogen is used as a fuel. In the semiconductor industry, hydrogen is utilized either as an etchant or reducing agent, for instance in the fabrication of silicon-based nanostructures,^7,8^ the reduction of RuO_*x*_, and etching of Sn inside extreme ultraviolet (EUV) lithography machines,^9,10^ the atomic layer deposition (ALD) of various materials,^11^ and the cleaning of III–V semiconductors surfaces.^12^ Hydrogen possesses a low molecular weight and high chemical reactivity, making it an attractive candidate for such applications. However, its propensity to damage materials poses a threat to many of its applications.^2,13,14^ Therefore, understanding the interaction of hydrogen with surfaces is critical for (further) development of its applications.

Typically hydrogen absorption in a material requires dissociation of hydrogen molecules (H_2_) into hydrogen atoms/radicals (H*).^15^ H* can diffuse into a material, causing embrittlement, blistering, stress build-up, interface defects, chemical erosion, and/or reduction.^16−19^ To protect materials in reactive H_2_ environments, coatings of materials with low hydrogen diffusivity—hydrogen permeation barriers—are used.^20^ Metals, metal oxides, nitrides, carbides, and graphene/graphite are reported as hydrogen permeation barriers.^21−23^ Permeation of hydrogen through these materials at elevated temperatures is well reported in the literature.^21,24−26^ Furthermore, because in fusion reactors, a significant wall flux of H* and ions occurs, numerous researchers have investigated the chemical erosion of carbon.^27−29^ Although hydrogen-induced chemical erosion of metals and their compounds is less likely to happen, H* can cause reduction of metal compounds that may degrade their barrier performance.^30,31^ While the potential damage to the material through hydrogen-induced metal–oxide, nitride, and carbide reduction is evident, the exact underlying mechanisms involved have not yet been extensively investigated.

The reduction of metal compounds is hypothesized to involve three steps: hydrogenation, formation of volatile species, and diffusion of subsurface atoms to the surface.^27^ Hydrogenation is influenced by the electronic structure of the host material.^32^ In the (simplified) model presented in this paper, it is assumed that H* in a material can absorb as an acceptor (H^–^ binding with the TM cation), donor (H^+^ binding with the anion), or an isolated interstitial impurity (H^o^). In the case of semiconductors, the model reported in the literature proposes that the corresponding absorption energies depend on the Fermi-level energy of the host material with respect to its vacuum-level energy (work function).^33,34^ According to DFT calculations by Van de Walle et al. in,^34^ when the work function of the host material is lower than ≈4.4 ± 0.2 eV, the H^–^ formation energy is smaller than the H^+^ formation energy. In the case of TiN, the work function of the (100) plane is reported to be 2.96 eV.^35^ As a result, calculations of the hydrogen absorption energies as H^–^, H^+^, and H^o^ on the TiN (100) surface are reported as, respectively, −0.16, +1.4, and +0.23 eV.^36^ This suggests a higher affinity for H* to form a bond with Ti compared to N on the TiN (100) surface. Although our thin films of transition metal nitride (TMN) are far from ideal single-crystal plane surfaces, we use this model to predict H* binding affinity in our TMN samples.

As a second step after H* adsorption, we assess the formation of volatile H-compounds with the TMN sample material. The energetic favorability for forming volatile species can be determined in terms of the difference between the Gibbs energies of reactants and products (Δ*G*). For example, under standard temperature and pressure, Δ*G* for denitridation of TiN by H* under the formation of ammonia (NH_3_) is calculated to be negative.^37^ However, H* are likely to preferentially form bonds with the Ti cations in TiN,^36,38^ making the formation of volatile NH_3_ less likely. Therefore, in this work, we investigate how the hydrogenation pathway defined by the work function and the thermodynamic favorability for forming volatile species influences the reduction of TMN thin films in a H* environment at elevated temperature (700 °C).

Understanding the interaction of H* with TMNs is necessary for evaluating their potential as protective coatings in reactive hydrogen environments. This knowledge may help to optimize process parameters in hydrogen plasma-enhanced ALD of TMNs^11^ or increase component lifetime from fusion reactors to EUV lithography equipment.

Therefore, the H* exposure temperature was specifically chosen for the results to be relevant to the development of TMN hydrogen permeation barriers in fusion reactors and EUV scanners where significant H* wall flux occurs.^28,39^ Hydrogen permeation barriers for fusion reactors are typically considered for the temperature range of [300–800 °C],^21,24^ while EUV scanner applications are reported to reach temperatures above 700 °C.^40^

## Material Selection

As inputs to the model presented in this paper, work function and thermochemical properties are required. Thus, we chose group IV and V TMNs (TiN, ZrN, HfN, VN, NbN, and TaN) based on the literature available regarding their hydrogenation, work functions, and thermochemical properties. For instance, Kura et al. experimentally validated hydric hydrogen defect (H–Ti groups) formation in TiN_*x*_ (0.7 < *x* < 1) and HfN_*x*_ (0.8 < *x* < 1) with no evidence for the presence of N–H states, arguing that the reason hydrogen acts as a shallow acceptor is due to the low work function of the host materials (<4.4 eV).^36,41^ Based on similar reasoning, Saito et al. in^42^ reported that hydrogen favorably form bonds with Zr cations in Zr_3_N_4−δ_. Analogous to experimental results, Bull et al. identified Ti, Zr, and Hf cations as the most favorable H* adsorption sites on (100) planes in TiN, ZrN, and HfN FCC crystals.^43^

The work function of the VN(111) surface is reported to be 4.39 eV,^44^ whereas for polycrystalline VN, the work function is expected to be lower.^45^ NbN and TaN surfaces exposed to air have work functions between 4.7 and 4.8 eV, slightly higher than ZrN and HfN surfaces after ambient exposure.^46^ Yet, the work functions of pristine NbN and TaN are expected to be greater than 4.6 eV.^45^ Nevertheless, TMNs are metastable and tend to form oxynitrides (TMO_*x*_N_*y*_) on the surface in ambient conditions, which increases their work function. The work functions of selected TMNs with native TMO_*x*_N_*y*_ surfaces are reported to be greater than 4.6 eV.^36,41,44−47^

## Hypothesis

**Figure 1:**
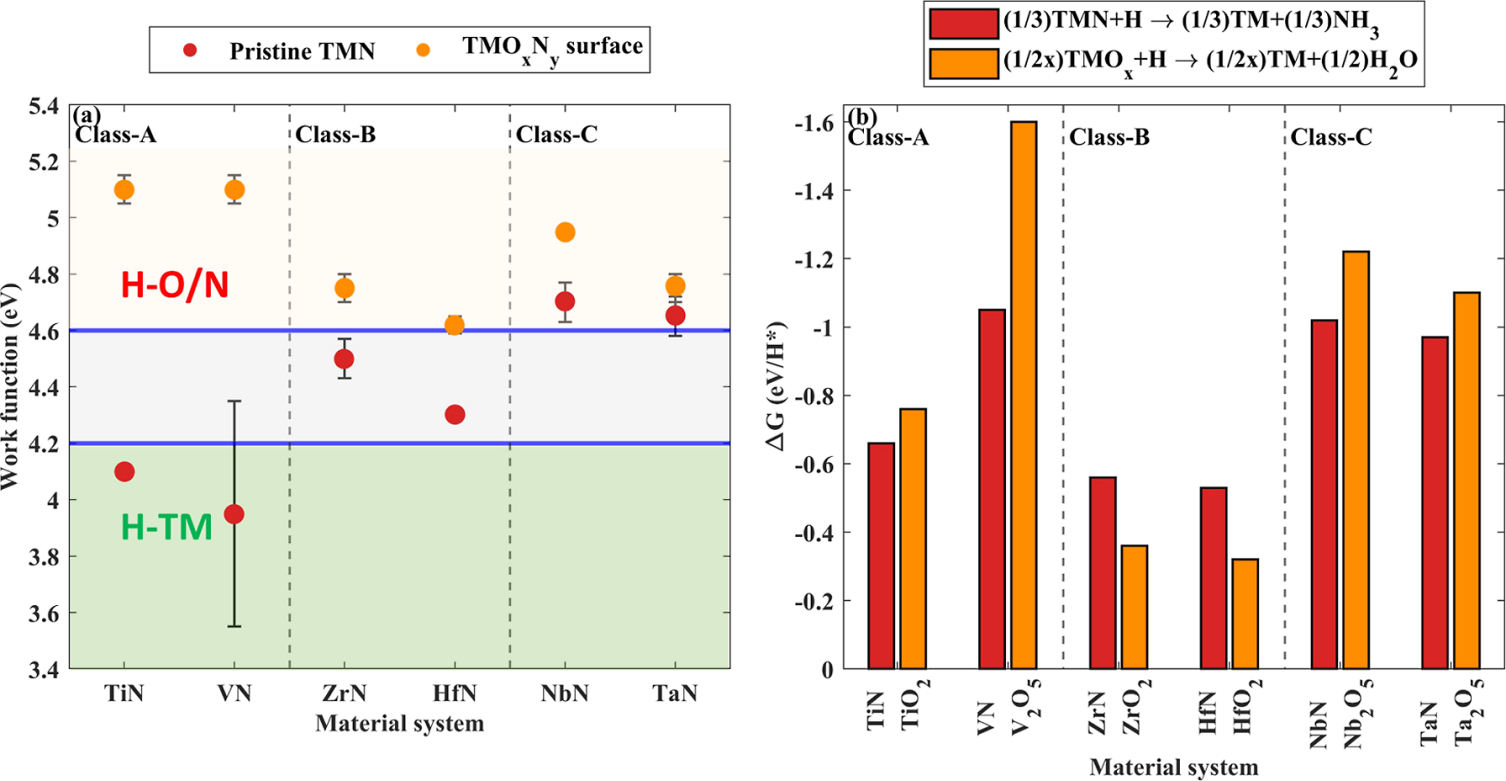
Reported work functions of pristine TMNs and their native TMO_*x*_N_*y*_ surfaces.^36,41,44−47^ (b) Δ*G* for removing N and O from pristine TMN and TMO_*x*_, respectively, per H* simulated at 1000 K. The work functions of TMO_*x*_N_*y*_ surfaces are greater than 4.6 eV; hence, the reduction is likely to occur. Based on Δ*G* values, it is apparent that for ZrO_*x*_N_*y*_ and HfO_*x*_N_*y*_ surfaces, denitridation is favorable and that for the other studied TMO_*x*_N_*y*_, layer deoxidation is more energetically favorable.

For predicting the interaction of H* with TMNs and their native oxynitride surfaces from ambient exposure, the model presented by Van de Walle et al. is extrapolated to TMNs and TMO_*x*_N_*y*_ to propose the most favorable H* absorption pathway (hydrogenation).^34^ The literature work functions of TMNs with and without the native TMO_*x*_N_*y*_ surfaces are shown in Figure 1a. With the exception of NbN and TaN, pristine TMNs have low work functions (<4.6 eV). Therefore, H* are expected to form bonds with the TM cations in pristine TiN, ZrN, HfN, and VN, making their reduction through NH_3_ formation less likely. However, the work functions of native TMO_*x*_N_*y*_ surfaces are greater than 4.6 eV, implying that H* are likely to form bonds with the O- and N-atoms near the surface. Since the work function is above the reported threshold, a reduction of the TMN/TMO_*x*_N_*y*_ surface is expected. The volatile species formed in this reduction process (H_2_O or NH_3_) will depend on the Gibbs energy of the reaction forming the volatile specie(s).

Figure 1b shows the Δ*G* values for the complete reduction of TMO_*x*_ and TMN simulated at 1000 K per H*.^37,48^ Since thermochemical properties of TMO_*x*_N_*y*_ are not well reported in the literature, the Δ*G* values for denitridation of TMN and complete deoxidation of TMO_*x*_ are used to determine the energetic preference for forming N-vacancy over O-vacancy on TMO_*x*_N_*y*_ surfaces. The Δ*G* values suggest that except for ZrO_*x*_N_*y*_ and HfO_*x*_N_*y*_, deoxidation of TMO_*x*_N_*y*_ surfaces is the energetically more favorable process (Figure 1b).

Three classes of TMNs are recognized based on their expected hydrogenation pathways (work function) and thermodynamic preference for forming volatile species. TiN, ZrN, HfN, and VN have low work functions (<4.6 eV). However, H_2_O formation is favorable for TiO_*x*_N_*y*_ and VO_*x*_N_*y*_ (class-A), whereas ZrO_*x*_N_*y*_ and HfO_*x*_N_*y*_ have a higher affinity to get denitridized by NH_3_ formation (class-B). Deoxidation of NbO_*x*_N_*y*_ and TaO_*x*_N_*y*_ is favorable; nevertheless, the work function of both pristine NbN and TaN is above 4.6 eV (class-C). Each class expresses a distinct behavior during H* exposures, which is discussed in the Results and Discussion section.

## Methodology

**Figure 2:**
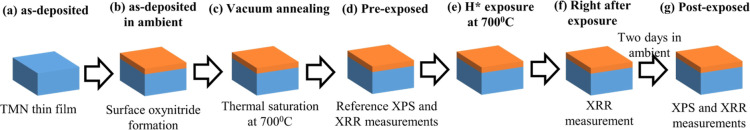
Schematic of methodology. Prior to H* exposure, as-deposited samples were annealed (pre-exp). XRR measurements were performed on the samples immediately following H* exposure (right after-exp). The samples were kept in ambient for two days (allowing surface reoxidation) before conducting XPS and XRR measurements (post-exp).

TMN thin films were deposited via reactive DC magnetron sputtering onto diced silicon wafer pieces of 15 × 15 × 0.5 mm with a 300 nm thick thermally grown SiO_2_ (Figure 2a). SiO_2_ serves as a diffusion barrier, in order to restrict the formation of metal silicides. The base pressure of the deposition chamber was in the low 10^–8^ mbar range for all depositions. Ar (99.999%) and N_2_ (99.999%) were used as sputtering gases with a flow rate of 15 sccm. The corresponding working pressure was set at 10^–3^ mbar. The thicknesses of the as-deposited films were intended to be 15 nm. This thickness was chosen to avoid interference from the processes that may proceed at the film-substrate interface during H* exposure. Grazing incidence X-ray reflectivity (XRR) measurements were performed using a Malvern Panalytical Empyrean laboratory diffractometer, which uses monochromatic Cu-Kα1 radiation to determine the thicknesses of the deposited layers. Deposition rates and thicknesses of as-deposited samples are listed in the Supporting Information (Table S1).

As-deposited samples were transferred via ambient to a Thermo-Fisher Theta probe angle resolved X-ray photoelectron spectrometer (AR-XPS), which uses a monochromatic Al-Kα source and a spot size of 400 μm. AR-XPS measurements revealed the formation of a surface TMO_*x*_N_*y*_ layer of a few nm thick on the as-deposited TMN thin film samples (Figure 2b).^49^ Samples were then vacuum annealed (max 10^–6^ mbar during anneal) for 2 h at 700 °C in order to saturate thermally induced processes before H* exposures are performed at 700 °C (Figure 2c). Samples were cooled to room temperature before breaking the annealing chamber’s vacuum to avoid excessive thermally activated surface oxidation.

AR-XPS measurements were performed on the annealed samples, where spectra were collected at take-off angles (ϕ) ranging from 26.75 to 71.75° with respect to the surface normal, resulting in probing depths ranging from approximately 1.5 to 5 nm. These annealed samples will be referred to as “pre-exposed” (pre-exp) samples (Figure 2d). XRR and AR-XPS measurements on pre-exposed samples were used as a reference in comparison to post-exposed samples to evaluate H*-induced changes. Additional XPS depth profile measurements were performed to measure the composition of the pre-exposed samples underneath the surface (sputter energy of Ar^+^ ions was set to 500 eV). The corresponding layer compositions are reported in the Supporting Information (Table S2). The at. % ratios between N and TM were found to be in the range of 0.7–1, yet all the samples are referred to as TMN in the text.

Pre-exposed samples were exposed to H* in a custom-built setup (Figure 2e). H* were generated by thermally cracking H_2_ via a W filament at approximately 2000 °C. The filament was positioned approximately 5 cm from the sample holder. The sample stage was heated to 700 °C via a radiative heater installed beneath the sample stage. The temperature of the stage was measured via Pt100 fitted inside the stage. The working pressure was measured to be 0.02 mbar. The H* flux on the sample surface was 10^21±1^ m^–2^ s^–1^.^50,51^ The samples were exposed to a fluence of H* at 7 × 10^24±1^ m^–2^, which is considered relevant to the industry.^39,52^ Before breaking the vacuum, samples were cooled to room temperature.

After ambient transfer, XRR measurements were performed on the samples immediately following the H* exposure, referred to as “right after-exposure” (Figure 2f). To allow reoxidation of the samples’ surfaces after H* exposure, the samples were stored in ambient for approximately two days. AR-XPS and XRR measurements were repeated on the samples after these two days. The corresponding samples are referred to as the “post-exposed” (post-exp) samples (Figure 2g).

The XPS spectra of pre- and post-exposed samples were compared to evaluate H*-induced stoichiometric changes. Over the range of AR-XPS measurements, spectra of individual samples showed similar variations. Hence, only the spectra collected at ϕ = 34.25° (and normalized to the maximum intensity) are discussed in detail in the Results and Discussion section, as they are more sensitive to the stoichiometry of the samples’ subsurface level (below 2 nm from the surface). The Supporting Information includes core level XPS spectra of TM collected at other ϕ (Figures S1–S3). To quantify denitridation upon H* exposure, the at. % ratio between N bonded to the TM and the TM itself was computed over the full range of AR-XPS measurements. Except for the TiN sample, the at. % of TM was estimated by calculating the area under the XPS spectrum of its core level and applying the relative sensitivity factor, to reduce ambiguities caused by fit quality. Due to the presence of satellite doublet (TiN-sat) in Ti 2p, TiN-, TiO_*x*_N_*y*_/TiO_2−δ_-, and TiO_2_-2p3/2, peak areas and sensitivity factor were considered to estimate the at. % of Ti in the TiN sample. The cumulative area under the TMN and TMO_*x*_N_*y*_ (and TMN_*z*_) peaks in the N 1s spectra along with the sensitivity factor were used to calculate the at. % of N in the sample. TMO_*x*_ fraction in the layers was estimated from the fitted TMO_*x*_ peak area in the core level spectrum of the TM.

Changes in layer thicknesses and densities were evaluated from XRR measurements of pre-exposed, right after-exposure, and post-exposed samples. For TiN, VN, NbN, and TaN samples, a bilayer model was used to fit the measured data: a thin TMO_*x*_N_*y*_ layer over a thick TMN layer; whereas, a single-layer model was used for fitting ZrN and HfN samples, as the second frequency was not apparent in the measured data due to low contrast at the interface. Density, roughness, and thickness were used as free parameters.

## Results and Discussion

**Figure 3:**
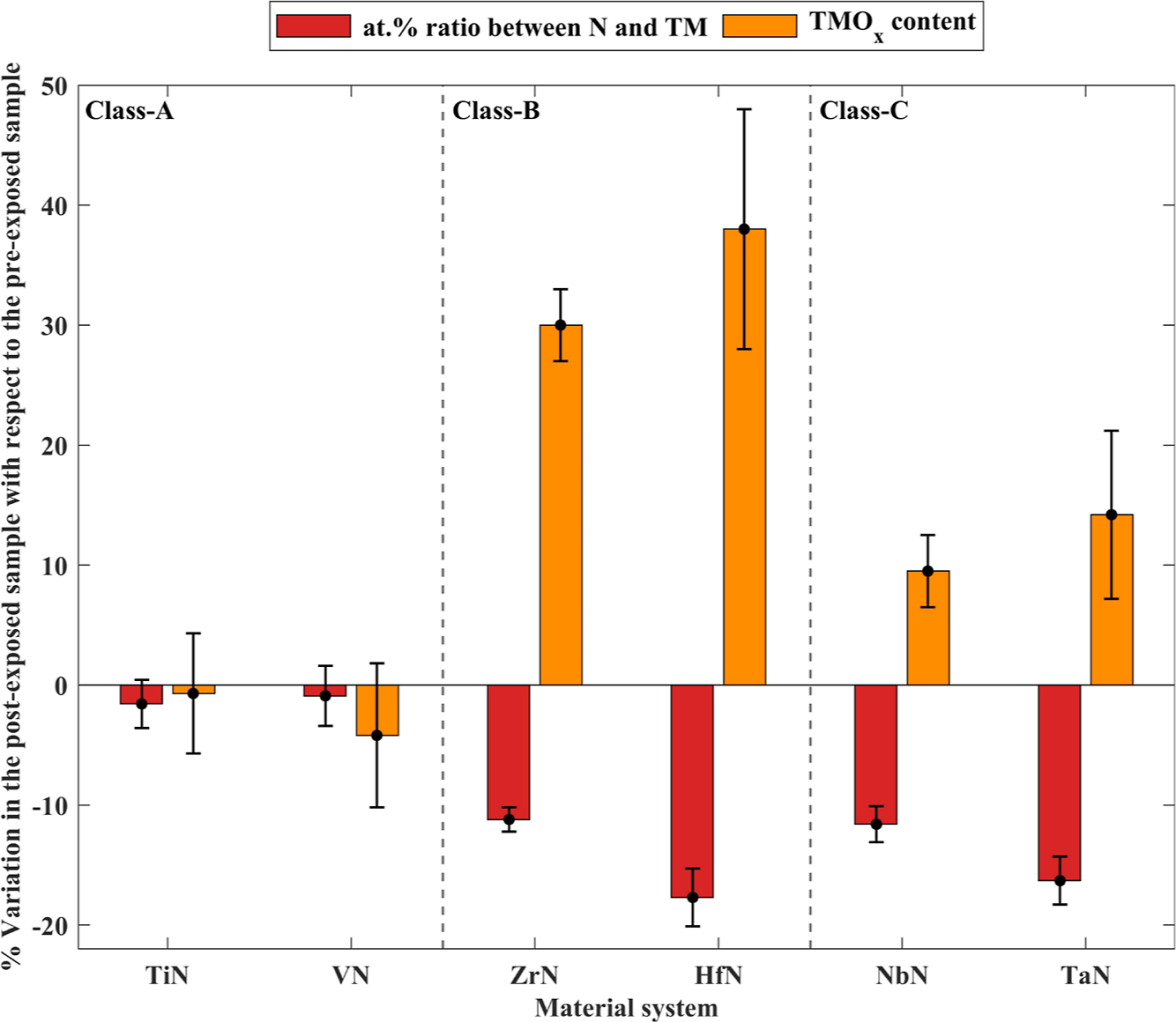
Observed denitridation and change in TMO_*x*_ fraction in the studied layers following H* exposure; expressed in terms of percentage variation in the at. % ratio from N to TM and the TMO_*x*_ content, respectively, in the post-exposed sample compared to the pre-exposed sample at ϕ = 34.25°. Variations in TiN and VN samples upon H* exposure were within the error margin of the measurements. Denitridation occurred in ZrN, HfN, NbN, and TaN samples. The increase in the TMO*x* fractions upon H* followed by ambient exposure in ZrN and HfN samples was substantially more than that observed in NbN and TaN samples.

In line with the hypothesis, the results of the studied layers are categorized into three classes based on their observed stoichiometry before and after H* exposures (Figure 3). In TiN and VN layers (class-A), change in the stoichiometry was insignificant. For ZrN and HfN samples (class-B), significant subsurface denitridation was found to take place, along with a strong increase in the TMO_*x*_ content. For NbN and TaN samples (class-C), a deep denitridation accompanied by a much lower increase in TMO_*x*_ content, as compared to samples of class-B was observed. The following subsections discuss each class separately.

### TiN and VN: Surface Oxynitride Reduction

**Figure 4:**
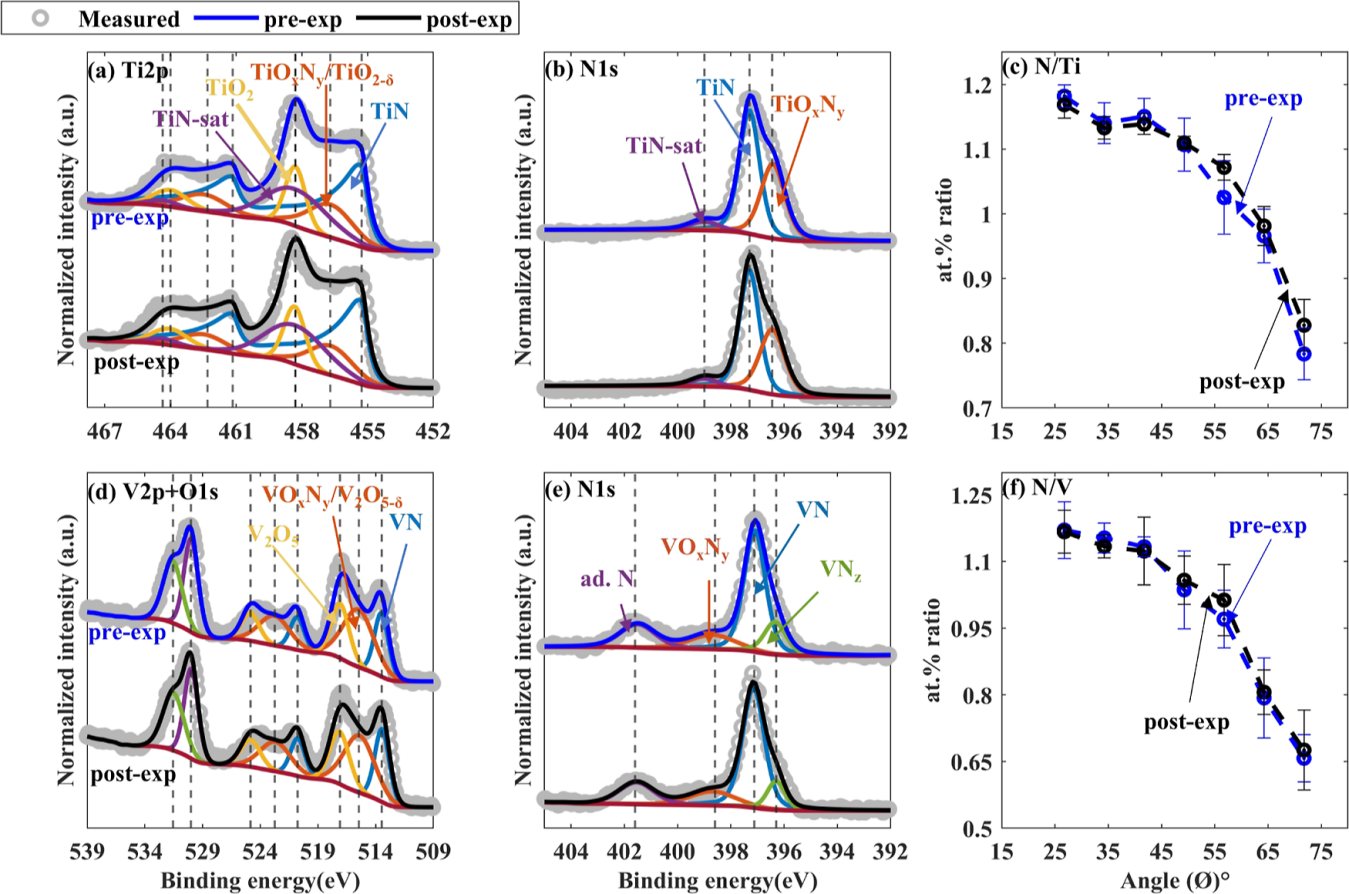
Pre-exposed (in blue) and post-exposed (in black) TiN and VN samples’ XPS spectra (collected at ϕ = 34.25°) and at. % ratio between N and TM as a function of ϕ. (a) Ti 2p, (b) N 1s of TiN samples, (c) N to Ti at. %, (d) V 2p, (e) N 1s of VN samples, and (f) N to V at. %. Variations in XPS spectra and N to TM at. % were found to be insignificant.

Figure 4 shows the XPS spectra of pre- and post-exposed TiN and VN samples collected at ϕ = 34.25° and N to TM at. % ratios as a function of ϕ. No shift in the positions of the peaks more than the measurement uncertainty (±0.2 eV) was observed in Ti 2p, V 2p, and N 1s spectra after H* exposure. Furthermore, variation in the spectral intensity at a given binding energy was insignificant, indicating that no change in the oxidation states of TM cations occurred (Figures 4a,b,d,e and SI1). Similarly, the variation in the N-to-Ti and N-to-V at. % ratios was calculated to be within the error margin of the quantification (Figure 4c,f). The XPS peak data are provided in the Supporting Information (Tables S3 and S4).

**Figure 5:**
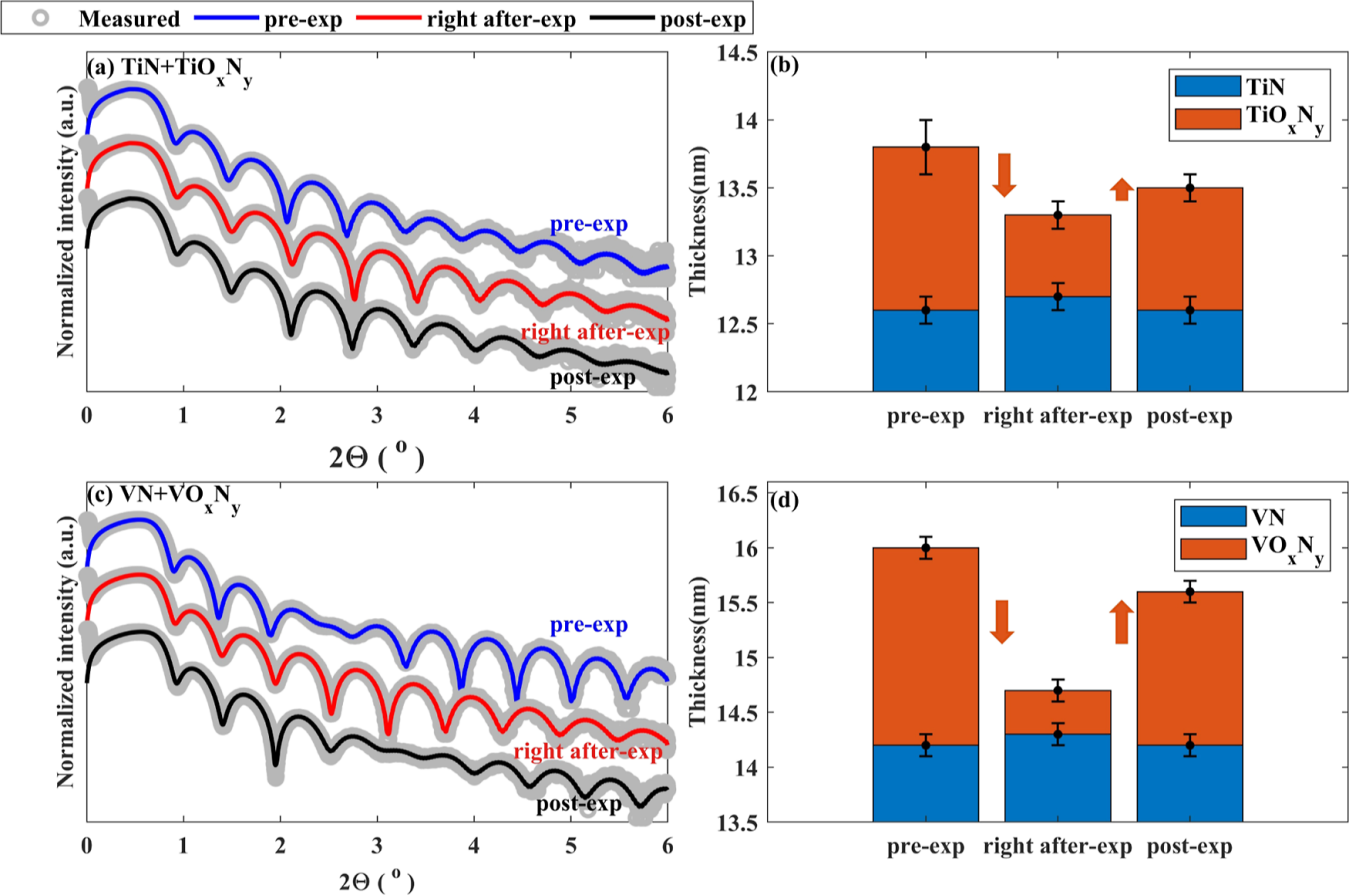
Measured and fitted XRR of pre-exposed (in blue), right after-exposure (in red), and post-exposed (in black) samples and corresponding fitted thicknesses. (a) XRR of TiN samples, (b) fitted thicknesses of TiO_*x*_N_*y*_ and TiN layers, (c) XRR of VN samples, and (d) fitted thicknesses of VO_*x*_N_*y*_ and VN layers. The smaller thicknesses of the TMO_*x*_N_*y*_ layers in the right after-exposure samples were due to the reduction of the surface oxides during H* exposure. Reoxidation in ambient caused an increase in thicknesses of the TMO_*x*_N_*y*_ layers in the post-exposure samples.

XRR data for pre-exposed, right after-exposure, and post-exposed TiN and VN samples along with fitted curves are shown in Figure 5. The fitted densities of TiN and VN layers were 4.9 ± 0.5 and 5.9 ± 0.5 g/cm^3^, close to the literature values.^53,54^ Changes in the fitted densities from pre-exposed to post-exposed samples were within the measurement error. However, frequencies of the fringes changed significantly, indicating variations in the thicknesses of the layers (Figure 5b,d). The thicknesses of the top TMO_*x*_N_*y*_ layers in TiN and VN samples decreased upon H* exposure by 0.6 ± 0.3 and 1.4 ± 0.2 nm, respectively. Upon exposure to ambient, the thicknesses of the top TMO_*x*_N_*y*_ layers were found to increase by 0.3 ± 0.2 and 1.0 ± 0.2 nm in TiN and VN samples, respectively.

Even though a significant change in the XPS of pre- and post-exposed TiN and VN layers is absent, XRR measurements on the samples immediately after H* exposure (right after-exp) suggest H*-induced changes in the samples within the surface level. Since N loss during H* exposure would be evident in the XPS signal, the decrease in the thicknesses of TMO_*x*_N_*y*_ layers upon H* exposure is likely to be related to oxide reduction, which is also in agreement with the proposed hypothesis. Furthermore, it is expected that during H* exposure, due to O loss and/or diffusion of subsurface N to the surface, the stoichiometry of the surface shifts from TMO_*x*_N_*y*_ to TMO_*x*–Δ_N_*y*+δ_ (Δ > 0 and δ > 0) resulting in a decrease of the work function. After partial or complete removal of O atoms from the surfaces, the work functions of TiN and VN samples are expected to decrease below 4.6 eV,^36,45^ according to our proposed model halting the further formation of volatile species. An oxygen-deficient surface (TMO_*x*–Δ_N_*y*+δ_) is expected to reoxidize swiftly in ambient, resulting an increase in the thickness of the surface layer due to the stoichiometry shifting back to pre H* exposed state (TMO_*x*_N_*y*_).

### ZrN and HfN: Denitridation Followed by Strong Oxidation

**Figure 6:**
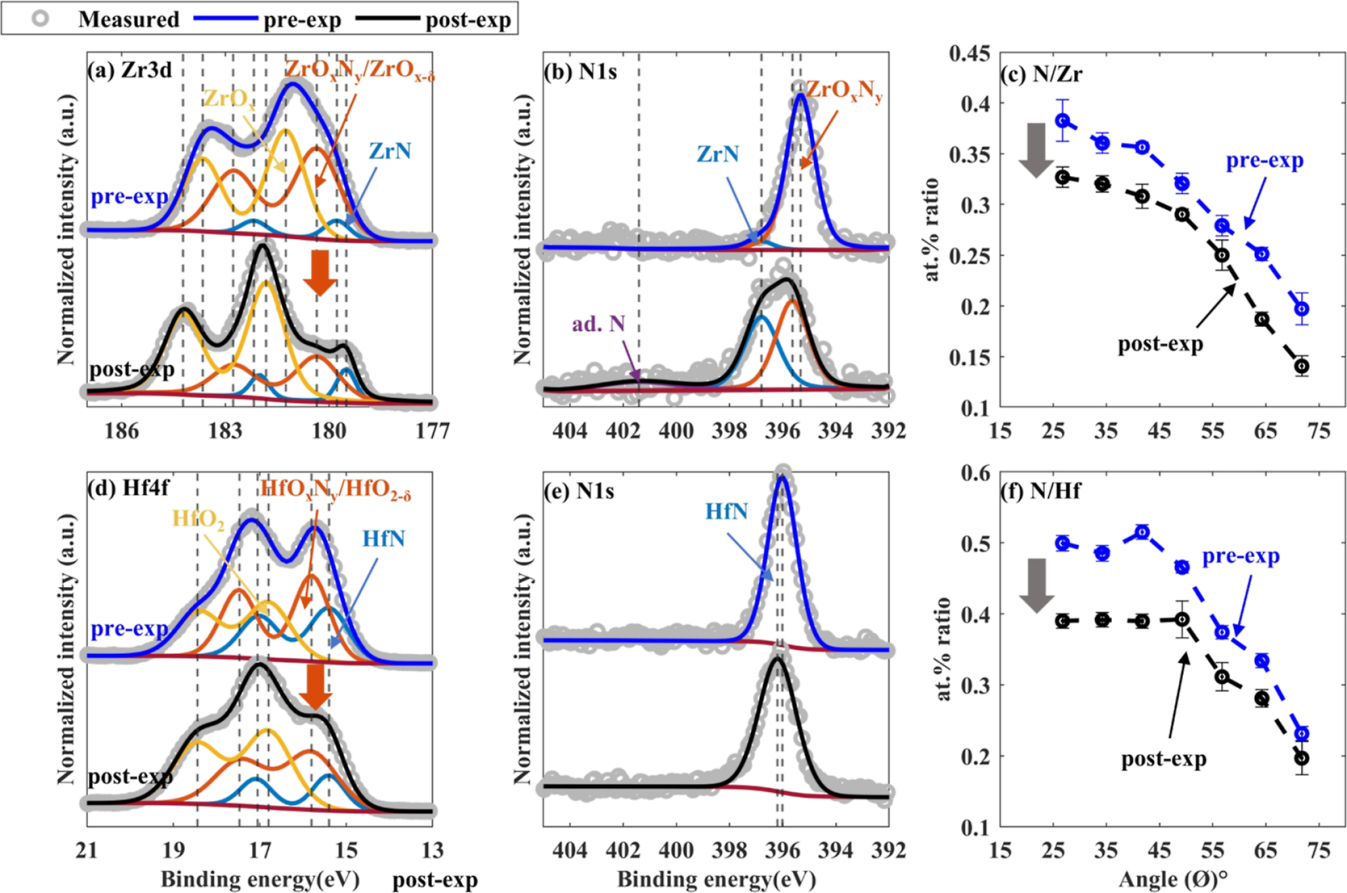
Pre-exposed (in blue) and post-exposed (in black) ZrN and HfN samples’ XPS spectra (collected at ϕ = 34.25°) and at. % ratio between N and TM as a function of ϕ. (a) Zr 3d, (b) N 1s of ZrN samples, (c) at. % ratio between N and Zr, (d) Hf 4f, (e) N 1s of HfN samples, and (f) at. % ratio between N and Hf. The post-exposed samples of ZrN and HfN showed lower TMN and TMO_*x*_N_*y*_ content and significantly increased TMO_*x*_ content.

Contrary to TiN and VN, ZrN and HfN samples showed substantial changes in their XPS spectra and N-to-TM at. % ratio following H* exposure (Figure 6). Intensities of Zr 4d and Hf 4f spectra decreased at the lower binding energies and increased at higher binding energies after H* exposure, indicating an increase in the oxidation state of TM cations after H* exposure (Figure 6a,d). The Zr–suboxide (ZrO_*x*_) doublet, ascribed at 181.2 eV (3d5/2) in the pre-exposed sample, shifted to a 0.6 eV higher binding energy after the exposure (Figure 6a).^55,56^ The associated ZrO_*x*_ content was noted to increase by 30 ± 3 at. % along with a considerable drop in ZrO_*x*_N_*y*_/ZrO_*x*–δ_ fraction (indicated by the arrow in Figure 6a). Likewise, after H* exposure, a substantial decrease in HfO_*x*_N_*y*_/HfO_2−δ_ content and an increase in HfO_2_ content (38 ± 10%) was observed in the HfN sample^57,58^ (Figure 6d). Over the range of ϕ, Zr 3d, and Hf 4f XPS spectra depicted a similar trend (Figure S2). An increase in the nitride content with respect to a decrease in the oxynitride content upon H* exposure in the HfN sample was not as pronounced as observed in ZrN (Figure 6b,e). However, a significant decrease in the at. % ratio between N and TM was noted in both samples (Figure 6c,f). Overall, the XPS results indicate that the nitride content was decreased and the oxide content was increased in the samples after H* exposure. Details regarding fitted peak positions are provided in the Supporting Information (Tables S5 and S6).

**Figure 7:**
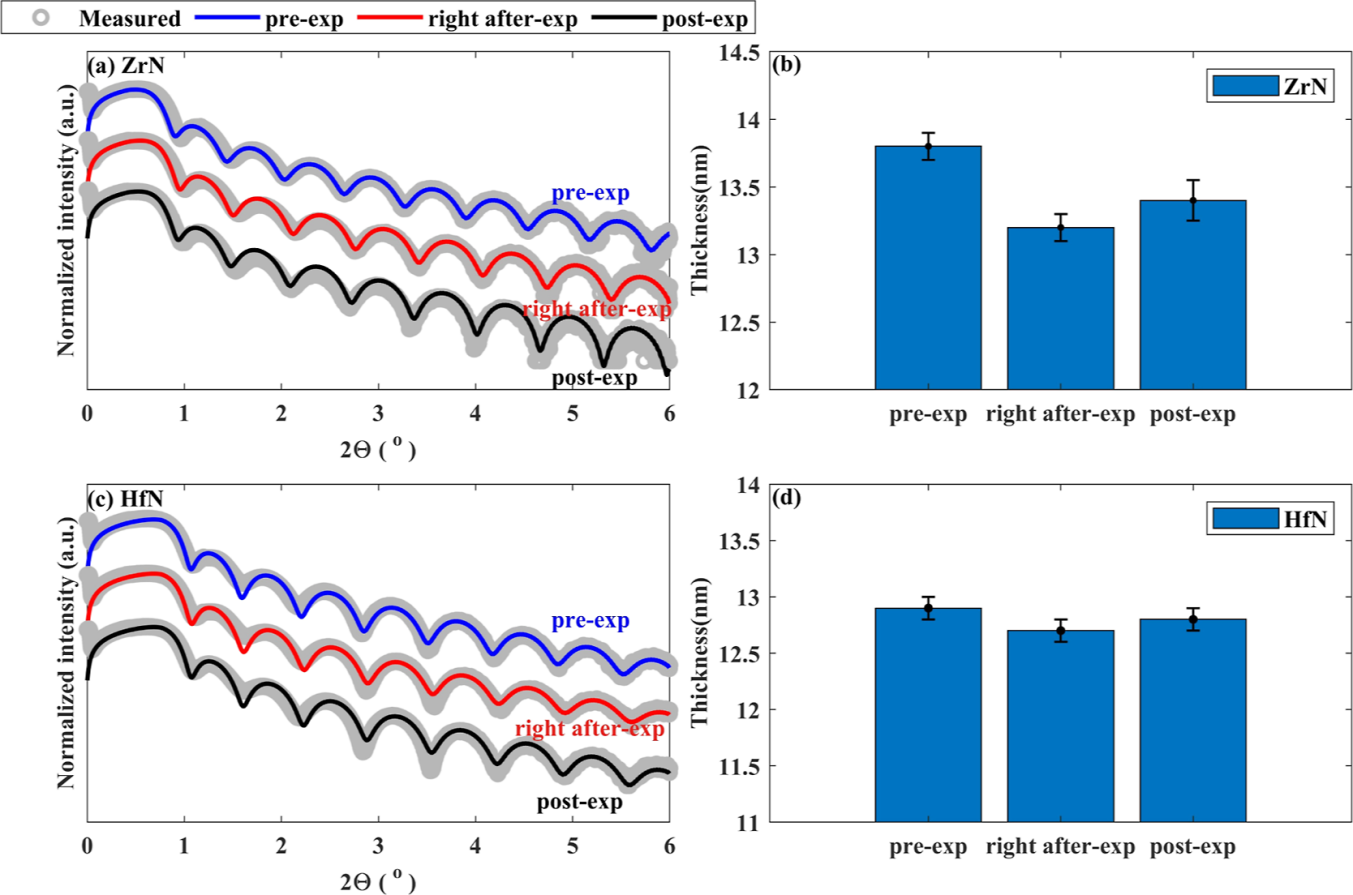
Measured and fitted XRR of pre-exposed (in blue), right after-exposure (in red), and post-exposed (in black) samples and corresponding fitted thicknesses. (a) XRR of ZrN samples, (b) fitted thicknesses of ZrN samples, (c) XRR of HfN samples, and (d) fitted thicknesses of HfN samples. The difference in the thicknesses of the pre- and post-exposed ZrN samples could be due to a higher ZrN fraction in comparison with ZrO_*x*_N_*y*_ content in the post-exposed sample. Variations in the thicknesses of the HfN samples were insignificant.

The measured and fitted XRR spectra of pre-exposed, right after-exposure, and post-exposed ZrN and HfN samples are shown in Figure 7. The fitted densities in the pre-exposed ZrN and HfN samples were 6.2 ± 0.5 and 10.5 ± 0.5 g/cm^3^, close to the literature values.^59^ Variation in densities after H* exposure and upon ambient storage was within measurement uncertainty. However, in the ZrN sample, a decrease in thickness (0.6 ± 0.2 nm) was noted immediately after H* exposure (right after-exp). The thickness of the sample increased in ambient (post-exp) after H* exposure by 0.2 ± 0.1 nm (Figure 7b). The difference in total thickness of the pre and post-exposed ZrN samples could be due to a relatively higher ZrN fraction compared to ZrO_*x*_N_*y*_ content in the post-exposed ZrN sample (Figure 7b). For HfN sample, no major change in the XRR spectra was found (Figure 7c,d). One reason for this might be that HfN has a higher affinity for absorbing O than ZrN.^60^ Nonetheless, further investigations are required to comprehend thickness changes in ZrN and HfN layers upon H* exposure.

According to the hypothesis, N vacancies were expected to be produced on the ZrO_*x*_N_*y*_ and HfO_*x*_N_*y*_ surfaces, which is consistent with the observations. It is likely that during H* exposure, N vacancies may easily be filled by diffusion of subsurface N atoms and the surface stoichiometry may only shift slightly from TMO_*x*_N_*y*_ to TMO_*x*_N_*y*–δ_. Hence, the work functions of the surfaces are likely to stay above 4.6 eV until considerable sample denitridation occurs. The higher TMO_*x*_ contents in the post-exposed samples are expected to be due to the enhanced oxidation during ambient exposure.

### NbN and TaN: Denitridation Followed by Weak Oxidation

**Figure 8:**
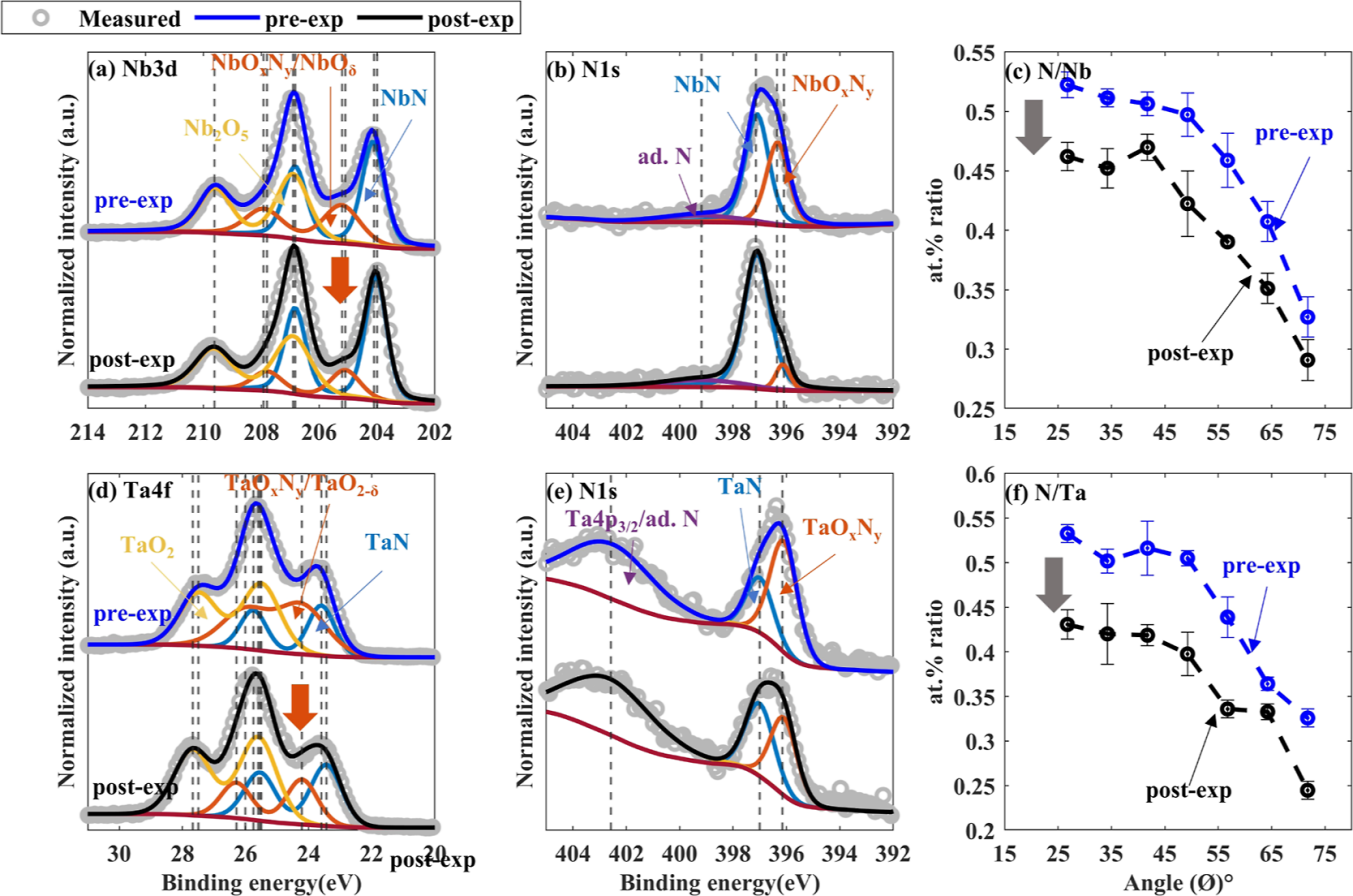
Pre-exposed (in blue) and post-exposed (in black) NbN and TaN samples’ XPS spectra (collected at ϕ = 34.25°) and at. % ratio between N and TM as a function of ϕ. (a) Nb 3d, (b) N 1s of NbN samples, (c) at. % ratio between N and Nb, (d) Ta 4f, (e) N 1s of TaN samples, and (f) at. % ratio between N and Ta. A decrease in the N to TM at. % ratio and an increase in TMO_*x*_ content suggested denitridation of the samples during H* exposure, followed by oxidation in ambient.

Similar to the ZrN and HfN samples, NbN and TaN samples showed substantial changes in the stoichiometry after H* exposure (Figure 8). In the post-exposed NbN and TaN samples, the intensity of NbO_*x*_N_*y*_/NbO_δ_ (δ < 2.5) and TaO_*x*_N_*y*_/TaO_2−δ_ doublets decreased, while Nb_2_O_5_ and TaO_2_ contents increased by 9.5 ± 3 and 14 ± 7%, respectively (Figure 8a,d).^61−65^ Over the range of AR-XPS measurements, similar variations were observed in Nb 3d and Ta 4f spectra (Figure SI3). The N 1s spectra of both the samples showed an increase in TMN content relative to TMO_*x*_N_*y*_ content after H* exposure (Figure 8b,e). Furthermore, the at. % ratios of N and TM in post-exposed samples were found to be lower than those in pre-exposed samples (Figure 8c,f). XPS results suggest a significant nitride reduction during H* exposure, while oxidation likely took place during sample storage in ambient. Tables S7 and S8 contain details of the fitted XPS peaks.

**Figure 9:**
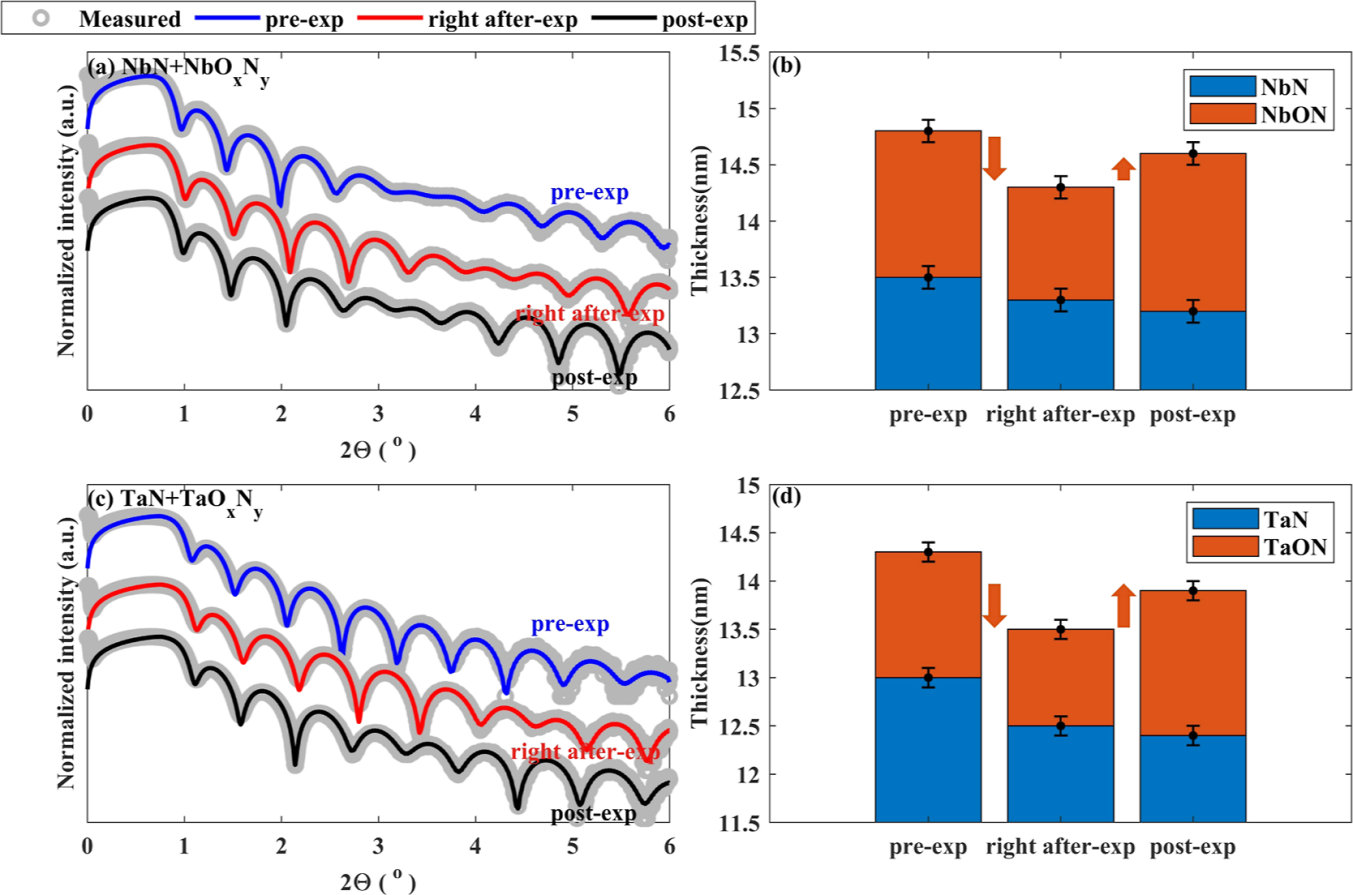
Measured and fitted XRR of pre-exposed (in blue), right after-exposure (in red), and post-exposed (in black) samples and corresponding fitted thicknesses. (a) XRR of NbN sample, (b) fitted thicknesses of NbN and NbO_*x*_N_*y*_ layers, (c) XRR of TaN sample, and (d) fitted thicknesses of TaN and TaO_*x*_N_*y*_ layers. Deoxidation of the samples followed by denitridation during H* exposure resulted in thinner TMO_*x*_N_*y*_ and TMN layers in the right after-exposure samples. An increase in the thicknesses of the TMO_*x*_N_*y*_ layers in the post-exposed samples suggested oxidation of H* exposed samples in ambient.

Figure 9a,c shows the measured and fitted XRR of pre-exposed, right after-exposure, and post-exposed NbN and TaN samples, respectively. The fitted densities of TMN layers in the pre-exposed NbN and TaN samples were 7.4 ± 0.5 and 12 ± 0.5 g/cm^3^, respectively.^66,67^ Variation in the fitted densities of TMN layers in the right after-exposure and post-exposed samples were within the measurement uncertainty. However, a drop in the thicknesses of the layers was observed right after H*-exposure (right after-exp). The thicknesses of TMN layers decreased by 0.4 ± 0.2 and 0.5 ± 0.2 nm in the NbN and TaN samples, respectively, due to subsurface denitridation during H*-exposure. In addition, the drop in the thicknesses of NbO_*x*_N_*y*_ and TaO_*x*_N_*y*_ layers by 0.3 ± 0.2 nm indicates partial or complete deoxidation of the surface oxynitride layers during the H* exposure (Figure 9b,d). Following ambient exposure, NbO_*x*_N_*y*_, and TaO_*x*_N_*y*_ layers’ thicknesses increased by 0.4 ± 0.2 and 0.5 ± 0.2 nm, respectively, due to oxidation. The overall thickness variation between pre- and post-exposed NbN and TaN samples were 0.2 ± 0.2 and 0.4 ± 0.2 nm, respectively. The thickness variation of the ZrN sample was also found to be in a similar range. Compared to the pre-exposed samples, the post-exposed NbN, TaN, and ZrN samples had significantly higher TMN content relative to the TMO_*x*_N_*y*_ fraction (Figures 6b and 8b,e), which could be one of the reasons for the thickness drop. However, more research is needed to understand the thickness changes caused by H* exposure.

The lower increase in TMO_*x*_ content (9–15%) in NbN and TaN samples after H* exposure compared to ZrN and HfN samples (30–38%) is explained by the deoxidation of the samples prior to denitridation during H* exposures, which is in agreement with the proposed hypothesis. It is expected that similar to TiN and VN samples, the stoichiometry of the surfaces of NbN and TaN samples change during H* exposure from TMO_*x*_N_*y*_ to TMO_*x*–Δ_N_*y*+δ_, however, the surface work functions are expected to stay above 4.6 eV.^45^ Thus, according to the proposed model, after deoxidation of the surfaces, the reaction should proceed with the formation of NH_3_.

## Conclusions

The chemical interaction of H* with ambient exposed TiN, VN, ZrN, HfN, NbN, and TaN thin films at 700 °C is reported. A model is proposed to explain the H*–TMN interaction that entails three steps: hydrogenation, formation of volatile specie, and diffusion of subsurface atoms to the surface. Based on the literature, the hydrogenation pathway is determined by the work function of TMN/TMO_*x*_N_*y*_. When the work function is less than 4.6 eV, H* form bonds with TM cations and, thus, reduction is less likely. However, as the work function increases above 4.6 eV, H* tend to form bonds with N and O atoms in TMN/TMO_*x*_N_*y*_. In this latter case, the energetic favorability for forming O- or N-vacancies is governed by Δ*G*. Due to vacancies on the surface, subsurface N atoms diffuse to the surface.

The H*–TMN interaction is categorized into three classes based on the proposed model and observations. We found that the reduction (deoxidation) in TiN and VN samples is limited to the surface (≈ top 2 nm). Subsurface denitridation along with a 30–38% increase in TMO_*x*_ content is noted in ZrN and HfN samples. Subsurface denitridation is also observed in NbN and TaN samples, however, the increase in the TMO_*x*_ fraction is 9–15%.

According to the proposed model, the formation of H_2_O is energetically favorable over NH_3_ on TiO_*x*_N_*y*_, VO_*x*_N_*y*_, NbO_*x*_N_*y*_, and TaO_*x*_N_*y*_. Thus, during H* exposure, the surface stoichiometry of the TiN, VN, NbN, and TaN samples shifts from TMO_*x*_N_*y*_ to TMO_*x*–Δ_N_*y*+δ_ with a drop in the work function. The work function of TiN and VN falls below 4.6 eV and no further reduction occurs. Whereas in NbN and TaN, the work function remains above 4.6 eV and the process continues with the reduction of nitrides following the reduction of oxides. On ZrO_*x*_N_*y*_ and HfO_*x*_N_*y*_, NH_3_ formation is energetically favorable over H_2_O. As a result of N diffusion from the thin film to the surface, the TM-to-N stoichiometry is restored. The reaction, hence, continues until substantial denitridation occurs.

## Supporting Information Available

Deposition rates and thicknesses, subsurface composition of the samples, core-level XPS spectra of TM as a function of ϕ, and fitted XPS peak positions (PDF)
