# Supplementary material for: Chemical Interaction of Hydrogen Radicals (H*) with Transition Metal Nitrides
Source: J Phys Chem C Nanomater Interfaces. 2023 Sep 6;127(36):17770–80. doi: 10.1021/acs.jpcc.3c04490 (PMC10510390; doi:10.1021/acs.jpcc.3c04490)
Supplement: Supplementary file 1 — jp3c04490_si_001.pdf [file jp3c04490_si_001.pdf]

Chemical Interaction of Hydrogen Radicals (H*) with Transition Metal Nitrides (TMNs)

Abdul Rehman[†*], Robbert W.E. van de Kruijs[†], Wesley T.E. van den Beld[†], Jacobus M. Sturm[†], and Marcelo Ackermann[†]

[†]*Industrial Focus Group XUV Optics, MESA+ Institute for Nanotechnology, University of Twente, Drienerlolaan 5, 7522NB Enschede, the Netherlands*

* E-mail: a.rehman@utwente.nl

Supplementary information

Table SI 1: Deposition rates and as-deposited thicknesses of TMNs.

**Material system**	**Deposition rate (nm s^{-1})**	**as–deposited thickness (nm)**
TiN	0.01	14±0.2
VN	0.05	16.3±0.2
ZrN	0.05	13.7±0.2
HfN	0.008	12.8±0.2
NbN	0.05	14.3±0.2
TaN	0.05	14.3±0.2

Table SI 2: Subsurface composition of pre-exposed TMNs.

**Material system**	**at.% TM (± 2%)**	**at.% N (± 2%)**	**at.% O (± 2%)**	**TM:N**
TiN	47.28	46.04	6.68	1 : 0.97
VN	50.88	42.97	6.15	1 : 0.84
ZrN	51.68	39.58	8.74	1 : 0.77
HfN	43.96	42.29	13.74	1 : 0.96
NbN	54	39.48	6.74	1 : 0.73
TaN	49.42	41.86	8.72	1 : 0.85

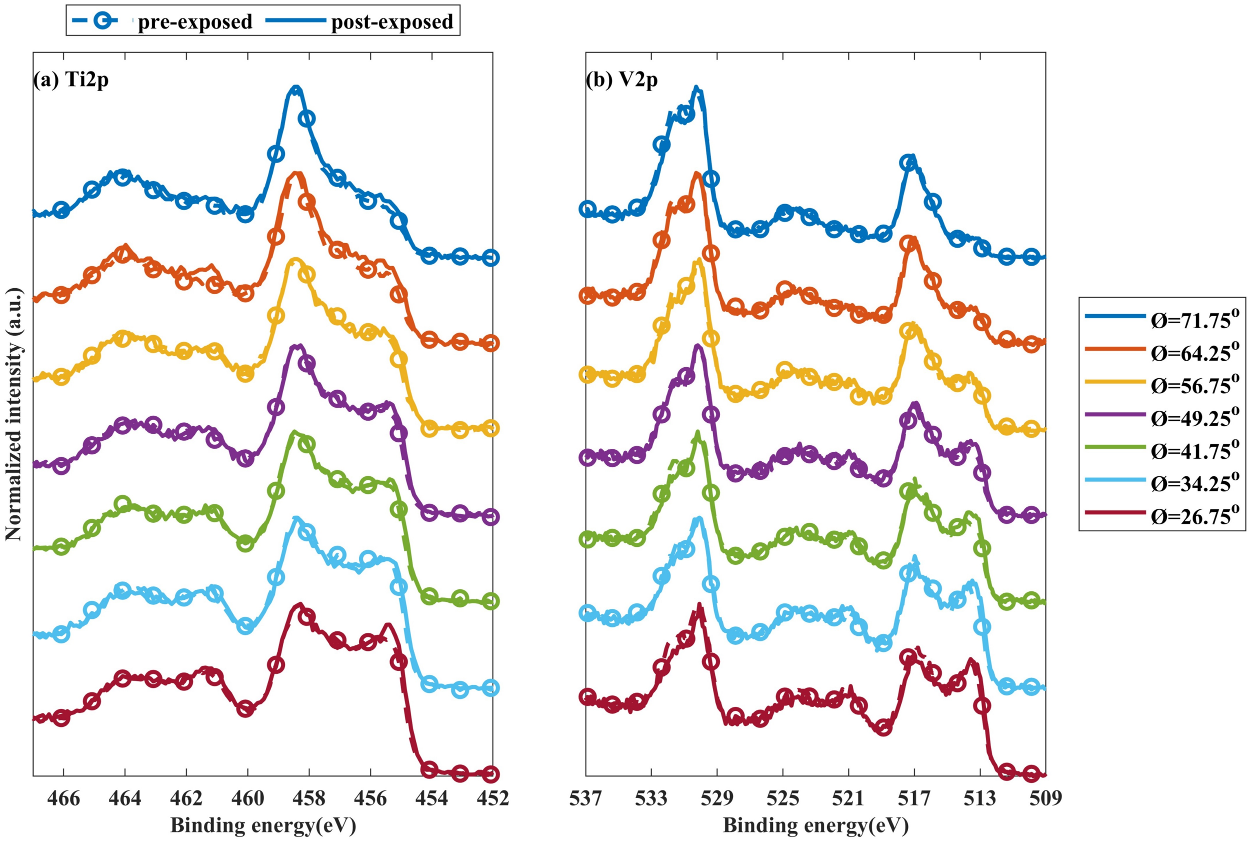

Figure SI 1: XPS spectra of pre–(with circular markers) and post–exposed (continuous line) TiN and VN samples at different ϕ. (a) Ti2p, (b) V2p. Variations in spectra were ascribed to be insignificant.

Table SI 3: Fitted peak position in per– and post–exposed TiN samples. Binding energies are referenced to the C-C peak at 285 eV. Ti2p spectra are deconvoluted into Ti2p3/2 and Ti2p1/2 with an average separation of 5.8 eV.

**Peak**	**Pre-exposed Binding energy (eV)**	**Post–exposed Binding energy (eV)**	**Reference**
TiN (Ti2p3/2)	455.3	455.3	455.3,[1] 455.5,[2] 455.03[3]
TiN-satellite (Ti2p3/2)	Ti2p3/2+3eV peak area=(Ti2p3/2)*0.16	Ti2p3/2+3 eV (peak area= (Ti2p3/2)*0.16	458.03 (3eV + 455.03),[3] 457.5[4]
$TiO_xN_y/TiO_{2-\delta}$ (Ti2p3/2)	456.7	456.7	456.8,[1] 457.2,[2] 456.59[3]
TiO_2 (Ti2p3/2)	458.3	458.3	458.3,[1] 458.4,[2] 458.18[3]
TiN (N1s)	397.3	397.3	397.3,[2] 397.23,[3] 397.2[4]
TiN-satellite/ $TiO_{x+\delta}N_y$ (N1s)	399	399	399.24(TiN–satellite),[3] 399($TiO_{x+\delta}N_y$)[2]
TiO_xN_y (N1s)	396.3	396.4	396.17[3]

Table SI 4: Fitted peak position in pre– and post–exposed VN samples. Binding energies are referenced to the C–C peak at 284.8 eV. V2p spectra are deconvoluted into V2p3/2 and V2p1/2 with an average separation of 7.5 eV.

**Peak**	**Pre-exposed Binding energy (eV)**	**Post–exposed Binding energy (eV)**	**Reference**
VN (V2p3/2)	513.5	513.4	513.5,[5] 513.9[6]
$VO_xN_y/V_2O_{5-\delta}$ (V2p3/2)	515.4	515.3	(515–517),[5] 515.5[6]
V_2O_5 (V2p3/2)	517	517.1	516.9[6]
VO_x/VO_xN_y (O1s)	530	530	529.9,[7] 530[8]
VO_xN_y/organic (O1s)	531.6	531.5	532.2[7]
VN (N1s)	397.1	397.1	397.1,[5] 397[6]
$VO_x + \delta N_y$ (N1s)	398.6	398.6	398.5,[5] 398.4[6]
VN_z (unsaturated N) (N1s)	396.3	396.3	396.5[5]

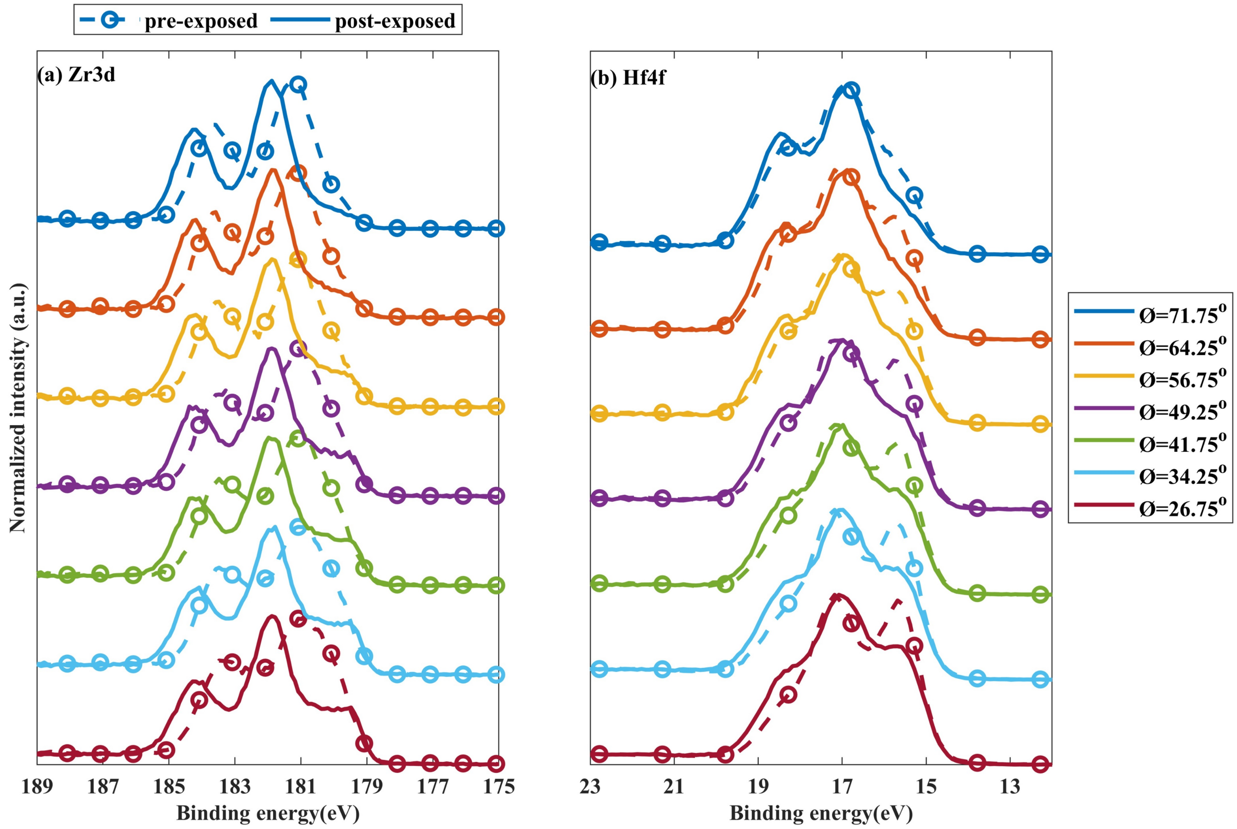

Figure SI 2: XPS spectra of pre–(with circular markers) and post–exposed (continuous line) ZrN and HfN samples at different ϕ. (a) Zr3d, (b) Hf4f. A decrease in the intensity at lower binding energies and an increase in the intensity at higher binding energies were regarded as denitridation upon H* exposure followed by enhanced oxidation upon exposure to ambient.

Table SI 5: Fitted peak position in per and post-exposed ZrN samples. Binding energies are referenced to the C-C peak at 284.6 eV. Zr3d spectra are deconvoluted into Zr3d5/2 and Zr3d3/2 with an average separation of 2.5 eV.

**Peak**	**Pre-exposed Binding energy (eV)**	**Post–exposed Binding energy (eV)**	**Reference**
ZrN (Zr3d5/2)	179.8	179.5	(179.3–179.7),[9] 179.2,[10] 179.9[11]
$ZrO_xN_y/ZrO_{x-\delta}$ (Zr3d5/2)	180.4	180.4	(180.8–181.56),[9] 180,[10] (180.8-181.4)[11]
ZrO_x (Zr3d5/2)	181.2	181.8	ZrO_2–(182.07–182.53),[9] ZrO_x–181.4[10]
ZrN (N1s)	396.8	396.8	(396.61–396.89),[9] 397.3[11]
ZrO_xN_y (N1s)	395.3	395.6	(395.34–395.95),[9] 396.2[11]
Ad N (N1s)	-	401.4	400.4[9]

Table SI 6: Fitted peak position in per and post-exposed HfN samples. Binding energies are referenced to the C-C peak at 285eV. Hf4f spectra are deconvoluted into Hf4f7/2 and Hf4f5/2 with an average separation of 1.6 eV.

**Peak**	**Pre-exposed Binding energy (eV)**	**Post–exposed Binding energy (eV)**	**Reference**
HfN (Hf4f7/2)	15.4	15.4	15.3[12]
$HfO_xN_y/HfO_{2-\delta}$ ((Hf4f7/2)	15.8	15.8	16.2[12]
HfO_2 (Hf4f7/2)	16.8	16.8	16.8,[12] 16.7[13]
HfN (N1s)	396.1	396.2	396.6[12]

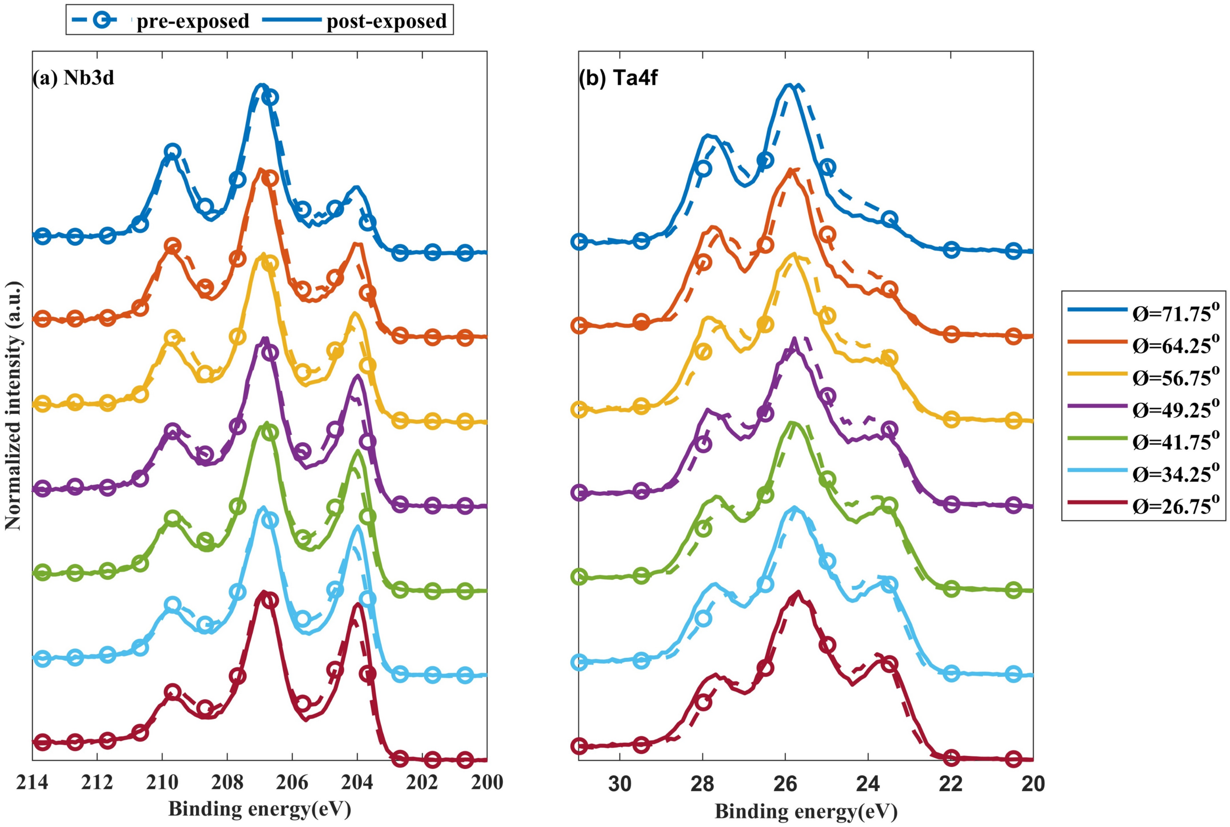

Figure SI 3: XPS spectra of pre–(with circular markers) and post–exposed (continuous line) NbN and TaN samples at different ϕ. (a) Nb3d, (b) Ta4f. A decrease in TMO_xN_y fraction along with an increase in TMO_x and TMN content was observed after H* exposure in both the samples. Overall N to TM at.% was found to be decreased after H* exposure.

Table SI 7: Fitted peak position in per– and post–exposed NbN samples. Binding energies are referenced to the C–C peak at 284.8 eV. Nb3d spectra are deconvoluted into Nb3d5/2 and Nb3d3/2 with an average separation of 2.8 eV.

Peak	Pre-exposed Binding energy (eV)	Post–exposed Binding energy (eV)	Reference
NbN (Nb3d5/2)	204.1	204	204[14] (203.76–204.25)[15]
$NbO_xN_y/Nb_2O_{5-\delta}$ (Nb3d5/2)	205.2	205.1	(204.77–205.76)[15]
Nb_2O_5 (Nb3d5/2)	206.9	206.9	207,[14] (207.21-207.27)[15]
NbN (N1s)	397.1	397.1	397,[14] (397.43–397.77)[15]
NbO_xN_y (N1s)	396.3	396.1	396.53,[15] (400–401.4)[14]
ad–N (N1s)	399.2	399.2	400[15]

Table SI 8: Fitted peak position in per–and post–exposed TaN samples. Binding energies are referenced to the C-C peak at 284.8 eV. Ta4f spectra are deconvoluted into Ta4f7/2 and Ta4f5/2 with an average separation of 2 eV.

Peak	Pre-exposed Binding energy (eV)	Post–exposed Binding energy (eV)	Reference
TaN (Ta4f7/2)	23.6	23.4	23.6,[16] $TaN_0.8$[17]
$TaO_xN_y/TaO_{2-\delta}$ (Ta4f7/2)	24.2	24.2	25.9[18]
TaO_2 (Ta4f7/2)	25.5	25.6	TaO_2-(25-25.3),[19] TaO-(23.5-23.9),[19] Ta_2O_5-(26.4-26.7),[19]Ta_2O_5-26.4[18]
Ta4p3/2 (N1s)	402.5	402.6	404.2[18]
TaN (N1s)	397	397	397.2[18]
TaO_xN_y (N1s)	396.1	396.1	396.6[18]

References

(1) Zorn, G.; Migonney, V.; Castner, D. G. Grafting titanium nitride surfaces with sodium styrene sulfonate thin films. *Biointerphases* **2014**, *9*, 031001.

(2) Eid, K.; Sliem, M. H.; Abdullah, A. M. Tailoring the defects of sub-100 nm multipodal titanium nitride/oxynitride nanotubes for efficient water splitting performance. *Nanoscale Advances* **2021**, *3*, 5016–5026.

(3) Greczynski, G.; Hultman, L. Self-consistent modelling of X-ray photoelectron spectra from air-exposed polycrystalline TiN thin films. *Applied Surface Science* **2016**, *387*, 294–300.

(4) Milošev, I.; Strehblow, H.-H.; Navinšek, B.; Panjan, P. Titanium nitride by XPS. *Surface Science Spectra* **1998**, *5*, 145–151.

(5) Osonkie, A.; Lee, V.; Chukwunenye, P.; Cundari, T.; Kelber, J. Plasma modification of vanadium oxynitride surfaces: Characterization by in situ XPS experiments and DFT calculations. *The Journal of Chemical Physics* **2020**, *153*, 144709.

(6) Li, S.; Sun, X.; Yao, Z.; Zhong, X.; Cao, Y.; Liang, Y.; Wei, Z.; Deng, S.; Zhuang, G.; Li, X. Biomass Valorization via Paired Electrosynthesis Over Vanadium Nitride-Based Electrocatalysts. *Advanced Functional Materials* **2019**, *29*, 1904780.

(7) Huang, K.; Bi, K.; Liang, C.; Lin, S.; Zhang, R.; Wang, W.; Tang, H.; Lei, M. Novel VN/C nanocomposites as methanol-tolerant oxygen reduction electrocatalyst in alkaline electrolyte. *Scientific reports* **2015**, *5*, 1–9.

(8) Mendialdua, J.; Casanova, R.; Barbaux, Y. XPS studies of V2O5, V6O13, VO2 and V2O3. *Journal of Electron Spectroscopy and Related Phenomena* **1995**, *71*, 249–261.

(9) Muneshwar, T.; Cadien, K. Comparing XPS on bare and capped ZrN films grown by

plasma enhanced ALD: Effect of ambient oxidation. *Applied Surface Science* **2018**, *435*, 367–376.

(10) Cubillos, G. I.; Romero, E.; Umaña-Perez, A. ZrN-ZrOxNy vs ZrO2-ZrOxNy coatings deposited via unbalanced DC magnetron sputtering. *Scientific Reports* **2021**, *11*, 1–19.

(11) Prieto, P.; Galán, L.; Sanz, J. Interaction of oxygen with ZrN at room temperature: an XPS study. *Surface and interface analysis* **1994**, *21*, 395–399.

(12) Piallat, F.; Beugin, V.; Gassilloud, R.; Dussault, L.; Pelissier, B.; Leroux, C.; Caubet, P.; Vallée, C. Interface and plasma damage analysis of PEALD TaCN deposited on HfO2 for advanced CMOS studied by angle resolved XPS and C–V. *Applied surface science* **2014**, *303*, 388–392.

(13) Barreca, D.; Milanov, A.; Fischer, R. A.; Devi, A.; Tondello, E. Hafnium oxide thin film grown by ALD: An XPS study. *Surface Science Spectra* **2007**, *14*, 34–40.

(14) Leith, S.; Vogel, M.; Jiang, X.; Seiler, E.; Ries, R. Deposition parameter effects on niobium nitride (NbN) thin films deposited onto copper substrates with DC magnetron sputtering. 19th Int. Conf. on RF Superconductivity (SRF 2019). 2019; pp 947–51.

(15) Havey, K.; Zabinski, J.; Walck, S. The chemistry, structure, and resulting wear properties of magnetron-sputtered NbN thin films. *Thin Solid Films* **1997**, *303*, 238–245.

(16) Arranz, A.; Palacio, C. Composition of tantalum nitride thin films grown by low-energy nitrogen implantation: a factor analysis study of the Ta 4 f XPS core level. *Applied Physics A* **2005**, *81*, 1405–1410.

(17) Valleti, K.; Subrahmanyam, A.; Joshi, S. V.; Phani, A.; Passacantando, M.; Santucci, S. Studies on phase dependent mechanical properties of dc magnetron sputtered TaN thin films: evaluation of super hardness in orthorhombic Ta4N phase. *Journal of Physics D: Applied Physics* **2008**, *41*, 045409.

(18) Cheng, J.; Xu, J.; Liu, L. L.; Jiang, S. Electrochemical corrosion behavior of Ta2n nanoceramic coating in simulated body fluid. *Materials* **2016**, *9*, 772.

(19) Moo, J. G. S.; Awaludin, Z.; Okajima, T.; Ohsaka, T. An XPS depth-profile study on electrochemically deposited TaO x. *Journal of Solid State Electrochemistry* **2013**, *17*, 3115–3123.
